# Current Developments and Role of Intestinal Ultrasound including the Advent of AI

**DOI:** 10.3390/diagnostics14070759

**Published:** 2024-04-03

**Authors:** Gennaro Tagliamonte, Fabrizio Santagata, Mirella Fraquelli

**Affiliations:** 1 Department of Pathophysiology and Transplantation, Università degli Studi di Milano, 20122 Milan, Italy; gennaro.tagliamonte@unimi.it (G.T.); fabrizio.santagata@unimi.it (F.S.); 2 Division of Gastroenterology and Endoscopy, Fondazione IRCCS Ca’ Granda Ospedale Maggiore Policlinico, 20122 Milan, Italy

**Keywords:** bowel ultrasound, intestinal ultrasound, elasticity imaging techniques, CEUS, SICUS, artificial intelligence, inflammatory bowel disease, Crohn’s disease, ulcerative colitis, point-of-care

## Abstract

Intestinal ultrasound is a non-invasive, safe, and cost-effective technique to study the small and large intestines. In addition to conventional B-mode and color doppler imaging, new US tools have been developed in more recent years that provide auxiliary data on many GI conditions, improving the diagnosis and assessment of relevant outcomes. We have reviewed the more recent literature (from 2010 onwards) on auxiliary tools in bowel ultrasound such as elastography techniques, CEUS, SICUS, and the potential contribution by artificial intelligence (AI) to overcome current intestinal ultrasound limitations. For this scoping review, we performed an extensive literature search on PubMed and EMBASE to identify studies published until December 2023 and investigating the application of elastography techniques, CEUS, SICUS, and AI in the ultrasonographic assessment of the small and large intestines. Multiparametric intestinal ultrasound shows promising capabilities in Crohn’s disease, while less is known about the role in ulcerative colitis. Despite some evidence, the CEUS role as a point-of-care examination tool for rare conditions such as intestinal GvHD and ischemic small bowel disease seems promising, possibly avoiding the need to perform further cross-sectional imaging. The use of AI in intestinal ultrasound is still anecdotical and limited to acute appendicitis.

## 1. Introduction

Despite advancements in technology, the initial evaluation and follow-up of small and large bowel diseases still relies on endoscopy, which has significant costs and still remains an invasive procedure that patients do not always accept well. Computed tomography (CT) and fluoroscopic studies are standard but involve high radiation doses. Thus, magnetic resonance and ultrasonography are preferred, especially for the assessment of chronic GI conditions (such as inflammatory bowel diseases), to minimize radiation exposure during repeated imaging. Intestinal ultrasound (IUS) is cost-effective, quick to administer, and allows for dynamic bowel evaluation, despite being considered highly dependent on the operator’s expertise [1]. In the last 15 years, the advancement of elastographic techniques and the availability of ultrasound-detectable contrast agents, along with the aid of artificial intelligence (AI), have increased the amount of data obtainable from the US evaluation of small and large intestines, broadening its application and relevance.

This narrative literature review covers the recent studies available on the new applications of transabdominal IUS auxiliary techniques such as ultrasound elastography, CEUS, and SICUS in the context of various non-neoplastic gastrointestinal diseases. Furthermore, it covers the current and future role of AI in IUS.

## 2. Materials and Methods

**Figure 1:**
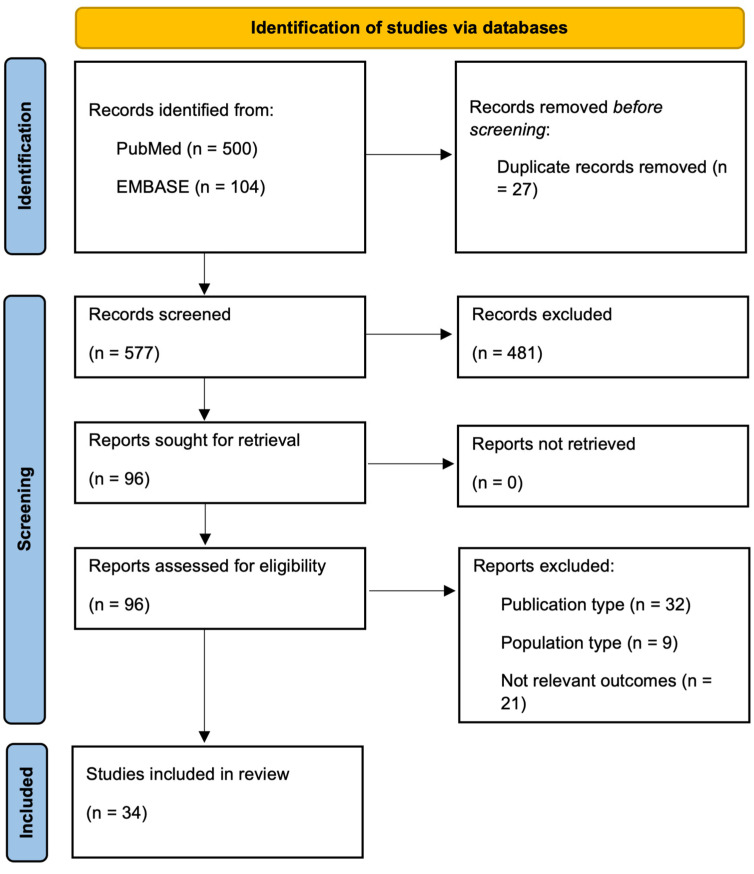
PRISMA flowchart showing the selection process of screened reports through systematic search.

An extensive bibliographical search was performed on PubMed and EMBASE to identify the literature published between 2010 and December 2023, using both medical subject heading (MeSH) terms and free-language keywords about the role of transabdominal IUS elastography techniques, CEUS, SICUS, and AI in the context of non-neoplastic gastrointestinal diseases. In total, excluding duplicates, 577 studies were screened for relevance. We found 96 potentially eligible studies of which we retrieved the full-text version. At the end, we identified 34 relevant studies. Further relevant studies not obtained from the systematic search were included. Conference abstracts and studies on pediatric populations were excluded (Figure 1).

## 3. Intestinal Ultrasound Technique

The ultrasonographic evaluation of the small and large bowels should be performed with both low-frequency (2–5 MHz) and high-frequency (5–17 MHz) linear array probes to provide a correct assessment of the bowel wall thickness and discrimination of the five different bowel wall layers (lumen/mucosa interface, mucosa, submucosa, muscolaris propria, serosa) [2]. As in all US examinations, intraluminal gas can represent a significant obstacle in obtaining good-quality images so the patient should be on fasting for at least 4 h before the exam and the gradual compression of the abdominal wall with the probe should be exerted during US evaluation [3]. Transabdominal IUS examination can be performed by identifying either the ileocecal region or the sigmoid colon in the right or left lower abdomen, respectively, using iliac arteries and veins as anatomical landmarks. No matter which course is taken, a full visualization of the colon is eventually achieved. The examination of the transverse colon can be difficult because of anatomical variability, while the rectum region cannot be adequately assessed with conventional IUS. Traditional grayscale imaging combined with a color or power doppler in IUS has a fundamental role in the assessment of many gastrointestinal conditions. In Crohn’s disease (CD) and ulcerative colitis (UC), ultrasonographic evaluation, at the start and follow-up, can detect pathological GI tracts (more commonly terminal ileum in CD) with typical wall thickening or changes in layer stratification, often associated with enlarged lymph nodes and mesentery hypertrophy around the bowel loops [4,5]. In order to correctly measure the bowel wall thickness, we should start from the inner hyper-echoic lumen/mucosa interface to the hyper-echoic serosa layer. By observing the alternating of the hyper- and hypo-echoic layers, the stratification pattern of the bowel wall can be assessed as preserved, partially disrupted, or disrupted with a prevalent hypo-echoic component. Moreover, the use of both grayscale imaging and CDI can detect the presence of CD-related complications such as strictures, fistulas, or abscesses [6]. Transabdominal ultrasound showed comparable high sensitivity to magnetic resonance enterography (MRE) in the assessment of intestinal inflammation and disease complications both in the context of CD and UC [7].

Many scores have been developed that correlate well with IBD disease activity or the clinical and endoscopic response to therapy, mainly using the bowel wall thickness (BWT), bowel wall stratification (BWS), and color doppler imaging (CDI) as parameters [8]. Despite this, the widespread adoption of IUS has been hampered by the lack of extensive validation of these scores. A recent study comparing the correlation of four differently validated US scores with endoscopy in CD found that IBUS-SAS outperforms other scores with the highest correlation with clinical activity (ρ = 0.58) and highest area under the curve (AUC) for any endoscopic activity (0.95 [95% CI 0.87–0.99]) [9,10]. In the context of UC, the only available validated index is the Milan ultrasound criteria (MUC), able to accurately discriminate active from non-active UC and predict a negative disease course with a cut-off value of 6.2 [11,12].

In contexts other than IBD, IUS plays a role in diagnosing or excluding acute appendicitis [13], demonstrating, when the appendix is correctly visible, a sensitivity of 71–92% and specificity of 94–100% for the diagnosis of acute appendicitis [14]. The inflamed appendix in sagittal images appears as a blind-ended, aperistaltic, non-compressible structure with an anteroposterior diameter greater than 6 mm, while in the transverse sections, it shows the typical “target sign” image [15]. Although partially underestimated, IUS also has a role in celiac disease; in the presence of clinical signs, the positivity of six US signs can confirm celiac disease diagnosis, while the lack of increased peristalsis and intestinal dilation confidently allows for excluding the presence of celiac disease [16].

## 4. Elastography Imaging Techniques

Ultrasound elastography represents an imaging technology that is sensitive to tissue stiffness, as first described in the 1990s. Elasticity imaging provides complementary information to conventional US by adding stiffness as another measurable property [17,18]. The current elastography techniques available with US devices used for small and large intestine assessment are either classified as strain elastography (SE) or shear-wave elastography (SWE). These techniques differ in the process used to calculate tissue deformation in response to applied pressure. SE calculates the tissue strain profile along the transducer axis after deformation and converts the strain profile to an elastic modulus profile. Tissue deformation can be induced by mechanical force that is either external (generated by pressing the US transducer against the tissue) or internal (using cardiovascular pulsation and/or respiratory motion). Furthermore, tissue can be deformed with acoustic radiation force impulse (ARFI), which targets ultrasound beam pulses to deform a chosen area. In SWE, tissue deformation induces shear waves perpendicular to the probe ultrasound beam. The shear-wave velocity is then measured to estimate tissue elasticity. The speed of the shear wave can be measured in a single point (point-SWE, pSWE) or at multiple locations, generating a color map of the shear-wave velocities in a discrete region of the tissue (2-dimensional-SWE, 2D-SWE). In the context of small bowel evaluation, pSWE has not been commonly used since it relies on a fixed region of interest (ROI), which often results in being too wide to only evaluate the bowel wall and exclude adjacent tissue. Shear-wave elastography (SWE) realizes a spatial map of the tissue elasticity, giving back a quantitative assessment of the stiffness measured as the Young’s modulus (kPa) [19]. Current evidence has shown possible advantages of SWE compared to SE in terms of operator-dependence, reproducibility, and reliability [20].

**Figure 2:**
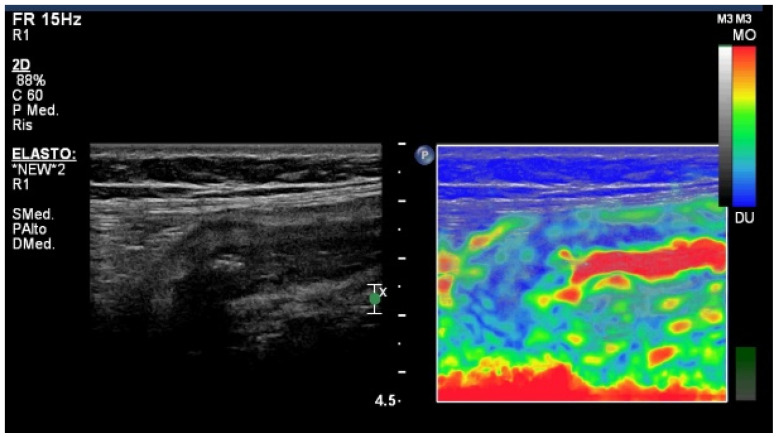
Intestinal ultrasound image showing a thickened terminal ileum with a prevalently stratified pattern (**left**) and color elasticity scale obtained with strain elastography SE (**right**).

**Figure 3:**
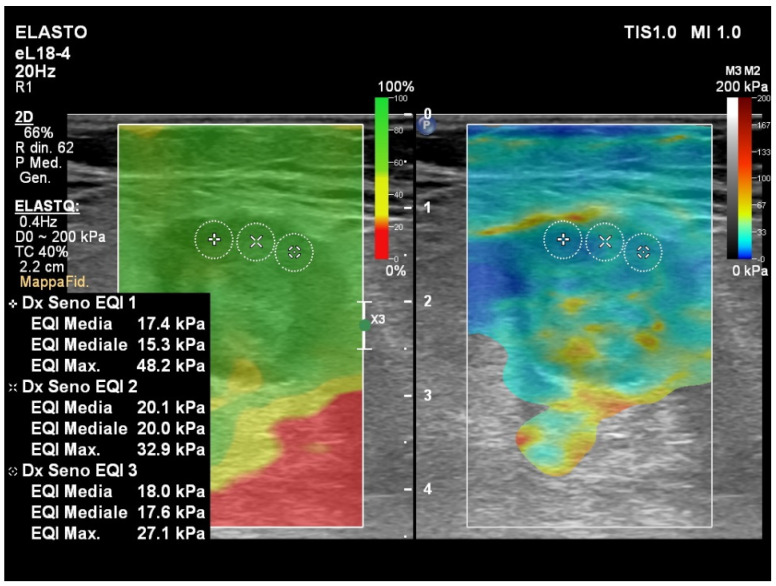
Intestinal ultrasound image showing a thickened terminal ileum with a prevalently hypo-echoic pattern at ultrasound shear-wave elastography (2D SWE) imaging.

The integration of elastography techniques in IUS can enhance the examination accuracy with information not obtainable from other cross-sectional imaging techniques, such as the tissue elasticity measurement. The main differences between the two most-used techniques, SE and 2D-SWE, are that SE relies on external compression, and cannot generate a quantitative measurement, but only a pseudo-quantitative measurement (strain ratio) by comparing the elasticity of the target area (target ROI) with a normal adjacent area (reference ROI) (Figure 2). On the other hand, SWE generates shear waves within the tissue using acoustic radiation force impulse (ARFI), providing quantitative measures of the elasticity stiffness by measuring the speed of these shear waves across the tissue (Figure 3). Recently, ARFI has been integrated in some strain imaging scanners with the aim of removing the need for external compression and possibly increasing reproducibility although there are still no conclusive data on this topic.

Both techniques have been applied in patients with CD to identify and grade mural fibrosis and to distinguish fibrotic from inflammatory strictures. Stricturing CD in its fibrotic or inflammatory subtypes represents the main application of ultrasound elastographic (US-E) techniques in clinical IBD management. Medical therapy has shown efficacy for the treatment of inflammatory but not fibrotic strictures [21], which can benefit from endoscopic dilations, surgical resections, or stricturoplasty depending on the extent of disease; hence, the importance of finding less invasive and more effective techniques to distinguish between these two clinical entities arises [22]. The histopathological analysis of the full bowel wall thickness is the current reference standard for the differential diagnosis of these two conditions [23]. It has been demonstrated that computed tomography enterography (CTE) features predict the nature of the wall thickening with good sensitivity [24,25]. 

Although not yet universally endorsed by international guidelines, elastography (whether SE or SWE) has demonstrated its efficacy in various studies in distinguishing between inflammatory from fibrotic strictures, as well as the response to treatment over time.

Evidence on SE highlights, in prospective studies, the correlation of the strain ratio measurement with the degree of fibrosis in histological specimens. Fraquelli M. et al. proved that the strain ratio measurement was significantly correlated with the severity of histological bowel fibrosis (AUROC 0.917; 95% CI 0.788–1.000) [26]; similarly, Baumgart DC. et al. found a correlation between SE and the histological evaluation of affected and unaffected CD bowel segments [27]. Statistical correlation was also found in comparing SE with other cross-sectional techniques, as Lo Re et al. proved when comparing the SE color scale and T2 signal intensity in stricturing CD to assess fibrosis levels [28]. Despite such evidence, some studies did not confirm the correlation between SE and fibrosis as reported by Serra C. et al. in a cohort of 25 stricturing CD, as no significant correlation was found between the strain ratio and fibrosis score (*p* = 0.877) [29].

The possible explanation for these differences in results mainly depends on two considerations: there is no stricture that is entirely fibrotic or inflammatory; thus, the ability of elastography to identify fibrosis falls in the presence of concomitant inflammation. To resolve this problem, it would be necessary to combine the evaluation with techniques that can discriminate inflammation with adequate sensitivity such as contrast enhancement, CDI vascularization, and SWD. Moreover, the quantification of elasticity with SE relies on the strain ratio calculation; therefore, there is a need to identify a reference ROI that is arbitrary and potentially influenced by CD activity, potentially invalidating the measurement.

The introduction of SWE has shown some promise in overcoming this issue; indeed, Ding. S. et al. evaluated suspected CD strictures with three different elastography techniques (SE, ARFI SE, p-SWE), finding that p-SWE had the best performance for evaluating and differentiating intestinal stenosis in CD, while neither SE nor ARFI imaging achieved satisfactory outcomes [30].

Several efforts have been made to demonstrate the accuracy of SWE in identifying fibrotic strictures by trying to identify a reproducible cut-off. Zhang M. et al. retrospectively compared computed tomography enterography (CTE) and 2D-SWE, along with the histopathology of the surgical specimen, of 37 patients with stricturing CD undergoing surgical resection: the stiffness value of 21.30 kPa measured with SWE was significant as a cut-off for identifying fibrotic lesions (AUC: 0.877, sensitivity: 88.90%, specificity: 89.50%, 95% CI 0.755~0.999, *p* = 0.000), and with a positive correlation of CTE in detecting inflammatory lesions (AUC 0.766, sensitivity 73.70%, specificity 77.80%). Combining SWE and CTE improved the diagnostic performance (AUC: 0.918, specificity: 94.70%) [31].

Similar results are reported by Chen Y.J. et al. on stricturing CD scheduled for surgical resection, comparing SWE with histological fibrosis: a cut-off value of 22.55 kPa could differentiate between mild/moderate and severe fibrosis, with the sensitivity and specificity, respectively, being 69.6% (95% CI 47.0~85.9%) and 91.7% (95% CI 59.8~99.6%) with an AUC of 0.822 (95% CI 0.685~0.960) [32]. Nevertheless, Lu C. et al. used SWE on 105 patients with ileal CD, finding a moderate correlation with histological muscular hypertrophy (*r* = 0.59), but not with inflammation or fibrosis; however, the shear-wave velocity was significantly lower in patients who did not undergo surgery compared to those who did undergo surgery [33]. 

Based on these findings, Kapoor A. et al. [34] have recently tried to validate shear-wave imaging (SWI) in patients presenting with chronic diarrhea and bowel wall thickening. They retrospectively evaluated a two-step diagnostic approach with IUS and SWI, first identifying inflammation or fibrosis on SWI (SWE cut-off of 22 kPa [31] for fibrotic lesions and an increase in SWD for inflammatory lesions). The subsequent step involved classifying the etiology of the disease based on greyscale IUS imaging. This approach predicted the underlying disease with an overall sensitivity and specificity combining SWE, SWD, and IUS of 100% and 99%, respectively. Furthermore, the results were compared with the CT findings for the prediction of bowel inflammation, highlighting the higher sensitivity of SWD as compared with CT (27% on CT vs. 100% on SWD) in detecting bowel inflammation [34]. These results seem very promising; however, it must be underlined that the retrospective nature of the study combined with the high prevalence of relatively rare conditions (CD and tuberculosis GI involvement) for patients presenting with common symptoms possibly suggests some sort of patient selection bias. On the other hand, the combined evaluation of shear-wave dispersion (SWD), used for the first time as a marker of inflammation, mirroring the approach used in hepatic shear-wave studies is enticing. Indeed, examining the dispersion characteristics of shear waves may indirectly offer insights into tissue viscosity, thereby providing biomechanical information regarding the pathological condition of the liver, such as necroinflammation [35].

The longitudinal assessment of tissue stiffness through elastography offers clinicians the ability to monitor disease progression and treatment response. Changes in tissue elasticity over time provide insights into the effectiveness of therapeutic interventions, allowing for timely adjustments to treatment plans. A current ambitious objective is to validate stiffness thresholds and changes in them during therapy as predictors of therapeutic outcome and markers of response in order to pursue a treat-to-target strategy. A recent study by Chen Y. et al. has prospectively evaluated multiparametric IUS (including SWE), as compared with endoscopy, in CD patients scheduled to initiate anti-TNF. The SWE value in the non-responsive group was higher than in the responsive group at baseline, and the cut-off value of 15.2 kPa could differentiate responders from non-responders with a sensitivity of 90%, specificity of 60%, and AUROC 0.775. In addition, SWE could discriminate non-responders better than BWT [36].

Similar evidence had been produced in SE by Orlando S. et al., proving a significant inverse correlation between the strain ratio values at baseline and the thickness variations following anti-TNF therapy, with a lower baseline SR predicting transmural healing [37].

Several authors have attempted to evaluate elastography in ulcerative colitis, achieving remarkable results. Yamada et al. compared SWE with endoscopy, detecting a significant correlation with SWE, but not SWD, with endoscopic and clinical disease activity, particularly in distinguishing patients who achieved mucosal healing from those in the active phase [38]. Goertz R.S. et al. compared the ARFI p-SWE of active UC with healthy volunteers, proving that overall, ARFI elastography was higher in the UC group than in the group of healthy volunteers (*p* < 0.021 and *p* < 0.001, respectively) [39].

A few studies are available on IUS elastography in benign non-IBD pathologies; some authors have further investigated the evaluation of acute appendicitis. The first study investigating the effectiveness of elastographic techniques in acute appendicitis was proposed by Kapoor A. et al., who analyzed a small cohort of patients with right lower quadrant abdominal pain where they found increased stiffness of the appendicular wall with SWE with high sensitivity compared to the histopathological examination of surgical specimens. This finding was also confirmed in the case of no US-detectable morphological alterations, successfully stratifying the condition severity on the basis of the area of increased stiffness circumferential extension [40]. Goya et al. found similar results, with ARFI imaging elastography showing increased wall stiffness in all cases of acute appendicitis. The stratification of the degree of inflammation according to stiffness was moderately correlated with the surgical findings (*r* = 0.539), proving to be a useful diagnostic tool especially when available clinical scores (Alvarado score) yielded an indeterminate result for diagnosis. Diagnostic confirmation was obtained through the histological examination of the surgical specimens [41]. Keven et al. demonstrated that the mean elastic modulus of an inflamed appendix was higher than that of normal ones. A cut-off of 23.2 kPa was found to be predictive of acute appendicitis with 97% sensitivity and 91% specificity in symptomatic patients [42]. Furthermore, in their study, Cha SW. et al. observed that the median elastic modulus of the appendix was markedly elevated in patients with appendicitis (25.0 kPa) compared to those without acute inflammation (10.4 kPa) [43].

## 5. Contrast-Enhanced Ultrasound (CEUS)

Contrast-enhanced US (CEUS) can enhance the representation of small bowel inflammation by demonstrating the rapid wash-in and slow wash-out of the microbubble contrast agent injected in peripheral veins. The contrast agent microbubbles (1–7 μm) are made of an inert gas (usually sulfur hexafluoride) encased by a phospholipidic membrane, their structure allowing them to oscillate at low frequencies around 3–5 MHz and through dedicated signal analysis generating a typical image. Small bowel ultrasound evaluation commonly utilizes higher-frequency probes, thus needing higher doses of contrast media to obtain the optimal signal quality [44].

**Figure 4:**
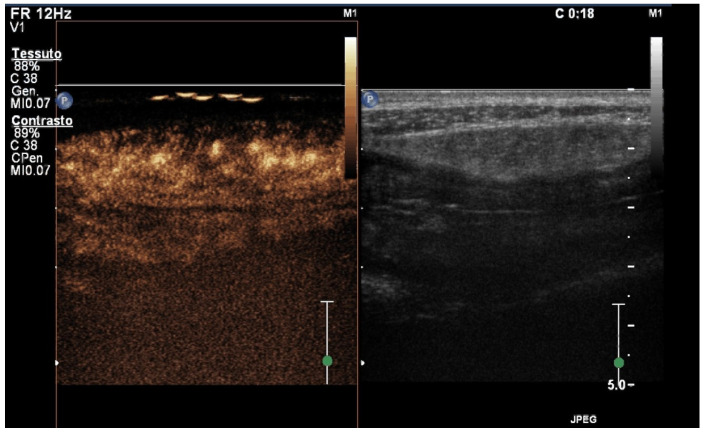
The terminal ileum presents a thickened and hypo-echoic bowel wall (**left**) with loss of normal layer stratification. Intestinal contrast-enhanced ultrasound (CEUS) image of the same tract showing thickened bowel wall and increased enhancement (**right**).

During IUS evaluation, after the injection of the contrast, the microbubbles travel from the peripheral vein through the pulmonary vascular bed to reach the bowel wall capillaries in about 10–20 s. After about 30–40 s, the concentration of microbubbles peaks (arterial phase) and then decreases (venous phase) thanks to the distribution in the capillary bed and veins, followed by the excretion of the contrast agent in the lungs [45] (Figure 4).

Traditionally, CEUS relies only on the qualitative analysis of the contrast media behavior. Dynamic CEUS (dCEUS) has been developed in order to provide for the quantitative analysis of US contrast agent kinetics.

There are two ways to generate quantitative estimates: the first one is called disruption–replenishment analysis, which relies on the continuous injection of the contrast for 5–20 min and the analysis of microbubble replacement kinetics after destruction with ultrasound waves. The second, more frequently utilized method is the wash-in/wash-out analysis, in which thanks to the data acquired during continuous registration after the bolus injection of the contrast agent, a time-intensity curve (TIC) is generated to estimate different measures such as the time to peak (TTP), peak intensity (PI), area under the curve (AUC), wash-in slope (P_w_), and mean transit time (MTT), all these different data being presented in log-normal to reduce the range of values and make them more manageable [46].

The recent update of the EFSUMB guidelines for CEUS in non-hepatic applications confirmed its role only in CD and in the assessment of vascular complications in the transplanted bowel or vascularity of gastrointestinal tumors [47]. Several preliminary studies have shown the potential for CEUS in recognizing predominantly fibrotic stenosis from inflammatory lesions in CD, although the data gathered to date are conflicting. Moreover, enhancement in the bowel wall layers correlates with clinical (assessed with the Crohn’s Disease Activity Index, CDAI) and endoscopic disease activity in CD [48]. A meta-analysis by Serafin Z. et al. on studies published until 2013 found a good CEUS pooled sensitivity (94%) and specificity (79%) for the diagnosis of CD activity [49].

Color parametric imaging (CPI) represents a recent development of CEUS: through image analysis software, color mapping is applied based on the differences in arrival times of the contrast media between the target region and a selected reference region. This allows for highlighting typical patterns of perfusion in lesions and has been shown to increase the diagnostic confidence of both residents and expert operators for the evaluation of liver focal lesions [50,51]. 

The performance of traditional B-mode US and CDI parameters to correlate with CD activity has been confirmed by multiple studies, and CEUS can significantly improve the detection of active disease. Freitas M. et al. compared the performance of CEUS against the Simple Ultrasound Activity Score for CD (SUS-CD) and the International Bowel Ultrasound Segmental Activity Score (IBUS-SAS) to correlate accurately with the terminal ileum endoscopic activity (assessed with the Simple Endoscopic Score for CD, SES-CD) in CD patients. While both SUS-CD and IBUS-SAS were not different between patients with active (SES-CD ≥ 7) or inactive (SES-CD < 7) endoscopic disease, the peak intensity (PI) with CEUS was significantly different. In the ROC curve analysis, the PI showed an AUC of 0.80 (95% CI 0.66–0.94). Moreover, Freitas M. et al. identified that a PI = 8.2 was the optimal cut-off value to correlate with active endoscopic disease, showing 74.1% sensitivity and 78.9% specificity [52]. In their study, Ma C. et al. compared multimodal ultrasound using 2D US, doppler US, CEUS, and elastography with endoscopy and laboratory markers in CD and UC. Both the PI and AUC parameters had a positive correlation with the endoscopy score, both in UC patients (*r* = 0.757, 0.693) and CD patients (*r* = 0.566, 0.571). Furthermore, TTP showed a strong negative correlation with the stenosis score (*r* = −0.754) while the PI and AUC showed a positive correlation with the stenosis score (*r* = 0.450, 0.643) [53]. Using multimodal IUS (with B-mode, color doppler, CEUS, and SWE), Jing J. et al. identified seven US parameters to be significantly different in active vs. inactive CD patients and established a regression model using CDAI as the reference standard for confirming the presence of disease activity. Among the different parameters, the texture of enhancement (TE) obtained by CEUS had the second-largest impact on the regression model [54].

The study by Statie R. et al. compared a combination of traditional IUS and CEUS with the resonance enterography (MRE) score (MaRIAs score) in CD patients. Both the combination of IUS/CEUS and MRE alone showed good accuracy in differentiating patients with active disease. However, the MaRIAs score showed a better correlation (*r* = 0.697) for fecal calprotectin levels and the Harvey–Bradshaw index (*r* = 0.594) [55]. 

Interestingly, Wilkens R. et al. found only a weak correlation between CEUS and the dynamic contrast-enhanced MRE (DCE-MRE) parameters in the small intestine of CD patients [56]. The same author also found the dCEUS parameters not to be significantly correlated to stricture stiffness in CD, while the DCE-MRE inflammation parameters showed a significant association with stiffness. These counterintuitive findings also confirm the possible role of inflammation and not only fibrosis in determining bowel stiffness [57].

Given the differences in the treatment of fibrotic or inflammatory stenosis in CD, the possibility of CEUS to distinguish these two entities is enticing; Ripollés T. et al. found that the percentage of increase in the contrast enhancement of the bowel wall shows a good performance as a predictor of inflammatory lesions (AUC 0.844), with a 65% increase in the enhancement cut-off showing 93% sensitivity and 69% specificity [58]. 

On the contrary, a negative correlation between the PI and fibrosis (*r* = −0.59) in CD patients was observed by Lu C. et al., who also found a significant correlation between the PI and histological chronic and not active inflammatory changes (*r* = 0.6) [33]. In assessing CD, the response to biological therapy is critical in order to avoid ineffective therapy continuation with potential side effects or disease progression. Many clinical trials have used CDAI as a therapeutic outcome, but it comes with the limitation of being based on patients’ symptoms while, in spite of being the preferred method, endoscopy has significant costs and low preference in patients. 

Earlier studies conducted in 2016 by Ripollés T. et al. found that multiparametric IUS (performed at 52 weeks from the start of anti-TNF therapy) significantly correlated with CD clinical response, but the researchers found no correlation between the increase in mural enhancement as assessed by CEUS and sonographic response or response to treatment [59]. The use of CEUS showed promise in subsequent studies on the early recognition of responders vs. non-responders to biological therapy in CD patients. In their prospective study, Quaia E. et al. found significant differences (assessed at week 6 of anti-TNF therapy) in the percentage changes in the peak enhancement (PE), wash-in rate, wash-out rate, and AUC correlated to better long-term endoscopic and clinical outcomes [60]. These findings were confirmed by the same authors in a later study on 115 CD patients, finding that both pre-treatment values and percentage changes in the PE and AUC after 6 weeks of anti-TNF therapy were correlated with better long-term outcomes [61]. Laterza L. et al. conducted a prospective study on 54 CD patients naïve to anti-TNF drugs with serial dCEUS assessment (at the starting point of IFX therapy, 2 weeks, 6 weeks, and 12 weeks of therapy) in order to identify key parameters able to differentiate responders from non-responders (as classified by endoscopy examination at 12 weeks) and to predict clinical relapse. A strong correlation was found between the early decrease (delta T1–T0) of four dCEUS: PI, AUC, P_w_, and MTT. In responders, the results remained consistent at T2 (delta T2–T0) and T3 (delta T3–T0) for all the considered parameters. Furthermore, the researchers also found that patients who relapsed within 6 months after IFX response showed a lower decrease in PI and P_w_ early (at 2 and 6 weeks) and an increase in both parameters at the 12-week evaluation [62]. Saevik F. et al., also using dCEUS, found a difference between responders and treatment failure in the PE, wash-in rate, wash-out rate, and wash-in AUC at 1-month US evaluation in a small group of 14 CD patients starting with either corticosteroids or anti-TNF [63].

Small intestine ischemic disease represents an insidious diagnosis requiring the combined use of CT, laboratory markers, and cautious clinical examination to correctly evaluate the need for surgery. Despite all this, only about a third of cases are correctly interpreted before explorative surgery. As demonstrated in a recent proof-of-concept study by Gummadi S. et al., the presence at CEUS examination of the simultaneous enhancement of the bowel wall relative to the surrounding tissue has a 100% NPV and an accuracy in predicting safe discharge without surgery of 87% against the 10% of CT [64]. As suggested by Gummadi S. et al., this places CEUS as an ideal rule-out test that potentially spares exploratory surgery in a good percentage of patients. In this context, CEUS is unlikely to replace CT but could benefit from the advantages of being a rapid, cost-effective examination carried out at bedside in unstable or critically ill patients.

Pausch A.M. et al. explored the role of false color-coded parametric imaging CEUS in the detection of acute gastrointestinal graft-versus-host disease (GvHD) compared to histology. After color coding the different arrival time of the contrast agent, two inexperienced operators evaluated the images, focusing on GvHD diagnosis in the presence of early arterial enhancement (shown as red and yellow area) and the transmural penetration of contrast media microbubbles (also color coded). The performance of parametric CEUS by the inexperienced operators was similar (89%) compared to the combined traditional B-mode US, elastography, and CEUS performed by experienced operators (95%) [65]. The parametric imaging CEUS potential has also been explored by Tews H. et al. in patients with severe COVID-19 disease to evaluate the presence of small bowel barrier disruption or microemboli. They found two subsets of patients, one showing early contrast enhancement in the bowel wall layers probably as an expression of systemic inflammation, and the other showing delayed contrast enhancement as an expression of microembolization-related hypoperfusion [66].

## 6. Small Intestine Contrast Ultrasound (SICUS)

Small intestine contrast ultrasound (SICUS) relies on small bowel distention to improve visualization through a macrogol solution as an oral contrast agent (about 250–500 mL). Repeated assessment is needed in order to evaluate the different tracts of the small bowel along with the contrast agent progression; thus, an examination can last up to 60 min. Compared to traditional grayscale US, in some studies on CD patients, SICUS has shown superior accuracy than standard US for the assessment of small bowel lesions [67] and disease complications (abscesses and fistulas) and the recurrence of disease following surgery. Furthermore, SICUS seems to reduce inter-observer and intra-observer variability by enhancing through dilation the appearance of pathological GI tracts in CD [68]. In celiac disease, SICUS can enhance typical US findings, allowing for both a more precise initial evaluation and assessment of response to gluten-free dieting (GFD) [69].

Despite the important role of intestinal ultrasound in first-time CD evaluation, the assessment of potential small bowel lesions is often suboptimal due to the presence of gas or the inadequate dilation of loops. The dilation of small bowel loops obtained with oral contrast in SICUS helps in the detection of CD lesions of the small bowel. The study by Onali S. et al. found a comparable high accuracy of computed tomography enteroclysis (CTE) and SICUS for detecting small bowel fistulae, strictures, and abscesses in CD [70]. In a recent meta-analysis, Zhu C. et al. found a good pooled sensitivity (88.3%) and specificity (86.1%), with an AUC of 0.9273 in detecting small bowel lesions for SICUS [71].

## 7. Artificial Intelligence (AI)

**Figure 5:**
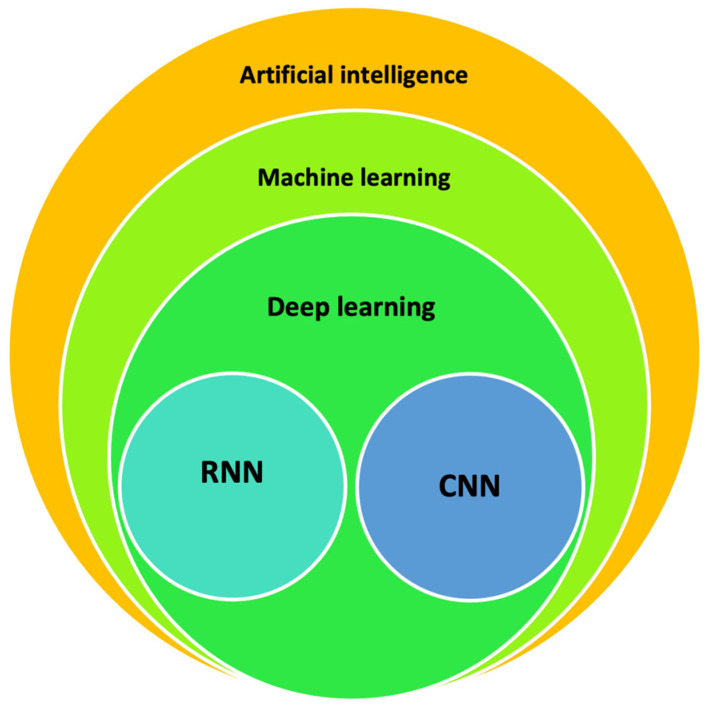
Euler diagram explaining the hierarchy of artificial intelligence (AI) technologies, RNN: recurrent neural networks, CNN: convolutional neural networks.

As AI continues to rapidly evolve in both everyday life and in the medical fields, there is a rising interest in its applications. AI represents an umbrella term that encompasses different aspects such as machine learning (ML) and specific techniques such as deep learning (DL) (Figure 5). The ML primary function involves the development of algorithms that can learn from and make predictions or decisions based on the provided data. Instead of being explicitly programmed to perform a task, these algorithms improve their performance on a task over time. ML encompasses various methods and techniques, including supervised learning, unsupervised learning, reinforcement learning, and deep learning. DL represents a subset of ML that makes use of neural networks composed of many layers to model complex patterns in data. The key advantage of DL is its ability to automatically learn representations from data without needing manual feature extraction from fed data as opposed to ML techniques [72]. Despite this, ML can still be useful for simpler tasks as it needs lower computational power, resulting in it being more competitive in terms of cost-effectiveness. Different kinds of neural networks can be used for specific purposes; convolutional neural networks (CNNs) are specifically designed to process pixel data and are commonly used in image recognition and processing, picking up patterns in spatial data. On the other hand, recurrent neural networks (RNNs) are primarily used for the analysis of sequential data (e.g., ECG data, EEG data, patient records over time) in order to pick up patterns and predict relevant outcomes.

The recognition of the potential role of AI in gastroenterology led to the first global summit on AI in gastroenterology and endoscopy held in 2019. That summit focused on the importance of a multi-disciplinary approach with specialists, industry, and regulatory institutions to develop new technologies for application in the clinical setting [73].

To date, there is still no definite role or consensus on the use of AI technologies in transabdominal IUS. Studies exploring the role of DL in other imaging techniques such as CT-enterography (CTE) in CD found that automated measurements of the small bowel were comparable to those performed by an experienced radiologist [74].

Only a few studies relevant to the topic have been found. Carter D. et al. developed an AI model using a convolutional neural network (CNN) to distinguish the thickened bowel wall on a dataset of 1008 US images evenly distributed between normal (BWT < 3 mm) and abnormal images (BWT > 3 mm). After a training phase on 805 images and a classification phase on 203 images, the network exhibited an AUC of 0.9777 for distinguishing the thickened vs. non-thickened bowel wall in the ROC curve analysis [75].

Kim K. et al. applied a fuzzy ART learning algorithm (an unsupervised neural network) in the critical phase of the appendix extraction process from US images in order to account for the below-satisfactory extraction rates of other algorithms. Medical experts’ opinion was used to judge successful appendix extraction from the image. Appendix extraction based on its morphological features proved highly accurate with 95% sensitivity, a major improvement as compared to other techniques such as 𝐾-means-based (67.5% sensitivity) and ART2-based (82.5% sensitivity). Moreover, it successfully recognized the possible presence of intra-abdominal fluid, thus overcoming the main limitation of existing techniques [76]. The same authors, Kim K. et al., also later developed two other learning algorithms using unsupervised learning neural networks focused on automatic extraction from US images of inflamed appendix: a self-organizing map (SOM) algorithm with correct extraction in 93.25% of cases [77] and Fuzzy C-Means (FMC) with 96.5% correct extraction [78]. While the SOM was found to be susceptible to the appendix shape for successful extraction, FMC showed an important reduction in the false negative cases of appendix extraction, with the only main limitation being the need for a training phase of the algorithm.

Ghareeb W. et al. compared an acute appendicitis AI predictive model developed using machine learning processing against the Alvarado score, US criteria, and combined Alvarado score and US criteria, using histopathology as a reference standard. They developed the AI model through the pre-processing of different data from many variables (e.g., US findings, patients’ characteristics, or clinical features) in order to be compatible with ML processing. Interestingly, the AI predictive model showed higher sensitivity (100%) and accuracy (97.9%) compared to all three other scores, while its specificity (80%) was higher than the Alvarado score but lower than the combined Alvarado score and US criteria (100%) [79].

Only five relevant studies were found regarding the current role of AI technologies, ML, and DL [69,70,71,72,73], highlighting a possible gap in the knowledge and future field of research. The relevance of these technologies in the diagnosis and follow-up of gastrointestinal diseases through upper and lower GI endoscopy, video-capsule enteroscopy, or cross-sectional imaging is growing at a fast pace. The advancements in AI technologies and their implementation in everyday IUS examination possibly have a role in improving the performance of inexperienced operators or in the context of rare scenarios of difficult interpretation. Furthermore, it will possibly lead to a significant reduction in inter-observer and intra-observer variability that often represents one of the main drawbacks of ultrasonographic assessment. Still, it must be noted that, in contrast with cross-sectional imaging techniques, US does not utilize fixed angles for the slicing of the selected area. This possibly represents a limitation to the extensive implementation of AI technologies for the automatic image recognition and detection of anatomical structures.

Data are lacking about the feasibility of real-time AI application in routine IUS examinations, which would potentially lead to the improved detection of pathological findings by showing an on-display indicator—as with some VCE software—to improve the operator detection of abnormalities. Moreover, the application of such software on handheld US devices, which have shown their reliability and agreement with traditional IUS [80], would dramatically improve the ability of clinicians in secondary centers to diagnose or follow up gastrointestinal diseases affecting small and large bowels, such as IBD and acute appendicitis. 

## 8. Conclusions

The current evidence suggests that in CD, the routine integration of both CEUS and US-E along with B-mode and color doppler imaging (CDI) is potentially critical in helping clinicians distinguish active from inactive ileal disease and assessing response to therapy. There is the need for a new tailored therapy strategy and for tools to achieve the tight evaluation of disease: IUS with its expanding range of capabilities is the ideal candidate.

New prospective studies on larger cohorts are needed to establish the exact role of multiparametric IUS assessment and the ideal timing to perform these examinations. On the other hand, there is still the need for defined cut-off values to stratify patient populations according to the type of lesion (fibrotic or inflammatory or mixed type) or the pattern of response to therapy. Moreover, US-E still suffers from the lack of standardization between different software and the unproven reproducibility of results among different operators. 

The role of both CEUS and US-E in UC is still not as widely explored; in theory, it would offer a smaller added benefit to established grayscale IUS and CDI assessment, given the prevalent mucosal nature of the disease. However, recent evidence shows that there is a potential role for CEUS to assess severe disease and response to therapy. On the other hand, the use of elastography in routine UC evaluation has been explored only on small cohorts of patients, suggesting some potential to assess disease activity.

The integration of CEUS in the evaluation of rarer gastrointestinal conditions such as GvHD, small bowel ischemic disease, and severe COVID-19 infection seems promising. In these scenarios, the value of IUS as a point-of-care examination to be performed rapidly in severely ill patients could avoid further examination by CT and MR techniques, avoiding significant costs, radiation exposure, and reducing the overall time to confirm a clinical hypothesis.

We have found only a small number of studies on the role of SICUS only in the context of CD, confirming the role of this technique in enhancing CD small bowel lesion detection, in particular for tracts of the small bowel that are difficult to assess with traditional IUS.

AI-based technologies are yet to be widely spread in IUS, while for comparison in endoscopy and cross-sectional imaging, their role is growing. This represents a gap in the knowledge and a possible area of future research. In our opinion, AI processing in IUS would possibly improve the performance of inexperienced operators, allowing for a quicker and better-quality examination. Moreover, it would possibly lead to a significant reduction in inter-observer variability that often represents one of the main drawbacks of ultrasonographic assessment. 

## Data Availability

No new data were created or analyzed in this study. Data sharing is not applicable to this article.

## Abbreviations

AI	artificial intelligence
ARFI	acoustic radiation force impulse
AUC	area under the curve
BWS	bowel wall stratification
BWT	bowel wall thickness
CD	Crohn’s disease
CDI	color doppler imaging
CEUS	contrast-enhanced ultrasound
CNNs	convolutional neural networks
CPI	color parametric imaging
CT	computed tomography
CTE	computed tomography enterography
DCE	dynamic contrast enhanced
DL	deep learning
GI	gastrointestinal
GvHD	graft-versus-host disease
IBD	inflammatory bowel disease
IUS	intestinal ultrasound
ML	machine learning
MRE	magnetic resonance enterography
MTT	mean transit time
PE	peak enhancement
PI	peak intensity
Pw	wash-in slope
RNNs	recurrent neural networks
ROI	region-of-interest
SE	strain elastography
SICUS	small intestine contrast ultrasound
SWD	shear-wave dispersion
SWE	shear-wave elastography
SWI	shear-wave imaging
TE	texture of enhancement
TIC	time-intensity curve
TTP	time to peak
UC	ulcerative colitis
US	ultrasound
US-E	ultrasound elastography
